# Peak alpha frequency is linked to visual temporal attention in 6-month-olds

**DOI:** 10.1038/s41598-024-79129-0

**Published:** 2024-11-15

**Authors:** Martina Arioli, Matteo Mattersberger, Stefanie Hoehl, Alicja Brzozowska

**Affiliations:** 1 Department of Psychology, University of Milano-Bicocca, Milan, Italy; 2 Department of Developmental and Educational Psychology, University of Vienna, Wien, Austria

**Keywords:** Peak alpha frequency, Temporal resolution of visual attention, Infancy, Neuroscience, Psychology

## Abstract

The temporal resolution of adults’ visual attention has been linked to the frequency of alpha-band oscillations in electroencephalogram (EEG) signal, with higher Peak Alpha Frequency (PAF) being associated with better visual temporal processing skills. However, relatively less is known about neural mechanisms underlying individual differences in the temporal resolution of visual attention in infancy. This study investigated the role of PAF in visual temporal processing in early infancy. In a sample of 6-month-old infants (*n* = 62) we examined the relationship between PAF extracted from resting-state EEG, and saccadic latencies in a predictive cueing task where the appearance of a reward was predicted by higher or lower frequency of two flickering objects. Results showed that higher PAF was associated with shorter saccadic latencies in a condition with higher differences between the two flickering frequencies, speaking for the involvement of PAF in visual temporal attention in early development. Additionally, we found that infants were generally faster to orient to the reward in trials where both peripheral stimuli were flickering at relatively lower frequencies, roughly corresponding to the theta frequency band. Our findings support theoretical accounts highlighting the role of PAF in visual attention processing and extend this framework to early infancy.

**Supplementary Information:**

The online version contains supplementary material available at 10.1038/s41598-024-79129-0.

## Introduction

Temporal resolution of visual attention determines how the visual system arranges dynamic perceptual information into coherent representation of objects, scenes, and events. This ability requires striking a balance between integration and segmentation processes to achieve an accurate understanding of the evolving visual environment. While the ability to integrate information over time is beneficial for generating a unified representation of the surroundings, it may also decrease sensitivity to detect rapid changes in the incoming sensory inputs. Therefore, for optimal efficiency, our visual attention must achieve a balanced interplay between these two processes^1^. Despite its importance for perception and cognition, little is known about the early development of visual temporal processing. The aim of this study is to elucidate neural mechanisms underlying individual differences in the temporal resolution of visual attention in infants.

In the typical population, the temporal resolution of visual attention improves across development. During the first year of life infants are more tuned to integrate visual information across time rather than segment it, with their Temporal Integration Window (TIW) – the period of time within which visual inputs are combined into a unique percept - being at around 190 ms between 6 and 12 months of age (reported in Freschl et al.,^2^). This window shortens rapidly during later infancy, reducing to around 140 ms at 18–36 months^3^ and reaching the adult-like level (i.e., 70 ms) by the age of 5 years^4^. Although the exact length of the windows differs, a similar developmental pattern was found by Farzin et al.,^5^, who compared 6-15-month-old infants’ and adults’ segmentation thresholds and found that the temporal resolution progressively shortens during development. Therefore, the visual attention system becomes more efficient in perceiving fast-paced visual changes across time through development, with a notable improvement occurring during the first years of life.

The temporal resolution with which infants scan the visual environment is crucial in their interactions with the surrounding world. Temporal integration and segregation of visual events are influential factors in determining the onset of different cognitive and motor processes, such as anticipating the trajectory of a moving stimulus to grasp it or binding synchronous temporal events (e.g., speech and lip movements) across space and different modalities. Fine temporal resolution is particularly important during early communication for the acquisition of language and turn taking skills that rely on synchronised timing between two or more individuals^6,7^. Furthermore, in toddlers, it is crucial for action sequence perception^8^ and in older individuals, for reading abilities^9^, multisensory integration^10^, action planning^11^, object file formation and visual working memory^12^. Therefore, disruptions in temporal resolution of visual attention can lead to cascading effects on different cognitive functions that rely on well-adapted timing for their computations. Consistent with this notion, recent research showed abnormalities in the temporal limit of visual attention in various neurodevelopmental conditions, such as fragile X syndrome^13^, dyslexia^9^ and autism^14^.

All this evidence suggests that accurate visual temporal processing is pivotal for the development of later cognitive and social abilities. Therefore, understanding the mechanisms underlying the temporal resolution of visual attention, as well as their neural substrates, is crucial for the development of early strategies to support visual processing abilities and mitigate potential cascading effects on later cognitive functions.

What are the factors influencing the temporal resolution of visual attention? According to the perceptual cycles theory^15^, perception is a discontinuous process, characterised by a series of discrete processing epochs. Our brain periodically samples information from the environment across different sensory modalities and organises these sensory inputs into a unique percept. There is a growing body of evidence that the sampling rate with which visual attention samples the sensory environment is determined by brain oscillatory activity, and in particular the alpha-band oscillation frequency. Alpha-band oscillations are the dominant oscillations in human scalp electroencephalogram (EEG), ranging between ∼7–13 Hz in adults and reaching their peak in parietal and occipital brain areas^16^. The frequency at which alpha oscillations show the maximum amplitude value is often called Peak Alpha Frequency (PAF; also known as Individual Alpha Frequency), and its role in visual attention sampling has been a topic of increased research interest in the last few years. Specifically, it was demonstrated that alpha-band oscillations actively contribute to segmenting visual inputs, with higher alpha frequency being associated with higher visual temporal resolution and higher perceptual accuracy. For example, Samaha and Postle^17^ showed that when two flashes occur within the same alpha cycle, then they are perceived as a single flash, whereas they are perceived as two different flashes if they happen in two different alpha cycles, thus suggesting that the speed of the occipital alpha rhythm of an individual determines their perceptual temporal resolution. Similarly, Venskus and Hughes^18^ demonstrated that higher Peak Alpha Frequency is associated with shorter Temporal Integration Windows. Interestingly, studies have demonstrated that altering the speed of alpha oscillations through transcranial alternating current stimulation (tACS) or sensory entrainment can either broaden or narrow the sensory integration window, thus influencing participants’ ability to detect discrepancy between two visual^19–21^ or audio-visual stimuli^22^, or modifying participants’ perceived frequency of visual stimuli^23^. Lastly, Tarasi and Romei^24^ demonstrated that the interindividual variability in PAF estimated during resting state predicted performances in a visual contrast detection task where participants had to detect a circle presented for a short period of time (i.e., 60 ms) in a checkerboard that varied in contrast. They found that individuals who succeeded in detecting the circle with lower contrast exhibited higher PAF compared to participants who required stronger contrast, thus suggesting that PAF is linked to sensory precision and perceptual accuracy.

Overall, these results suggest that PAF represents an index of individual temporal resolution of visual attention in adulthood. Developmental work so far has mainly linked PAF to broad non-verbal cognitive abilities in both autistic and typically developing samples^16,25–27^, without special focus on visual attention. However, indirect evidence suggests that the specific link between PAF and individual temporal resolution of visual attention might occur already at an early stage of development. A recent meta-analysis^2^ pointed out that PAF and visual temporal processing exhibit similar developmental trajectories, both reaching adult-like maturation around the age of five. Indeed, parallel to the improvement in temporal resolution of visual attention, average PAF undergoes rapid increases, rising from 6.1 Hz at 6 months to 8.4 Hz at 5 years, and stabilising during adolescence with an asymptote of 10.1 Hz at 18 years^2^ and 10.28 Hz at the age of 29 years^28^. Furthermore, additional evidence supporting the idea that PAF is involved in the early development of visual temporal processing comes from research on neurodevelopmental disorders, particularly autism, where higher PAF is typically observed^29^, along with shorter Temporal Integration Windows^14^. These findings suggest that PAF might be a driver for visual processing organisation across development. However, to the best of our knowledge, no study has yet directly tested the link between PAF and visual temporal processing in infancy.

*The current study*.

The present study aims to directly test the link between PAF and visual temporal processing in early infancy. To this aim, we used data collected in a large project examining neurobehavioral correlates of visual attention. Six-month-old infants’ EEG signal and looking behaviour were recorded during resting state and in a predictive cueing task, where the appearance of the location (left/right) of a reward (attractive moving cartoon character) was predicted by the higher or the lower of the two frequencies at which two identical objects were simultaneously flickering at the right and left side of the screen. The two objects flickered either both within the infant theta frequency band, both within the infant alpha frequency band, or one within the alpha range and the other within the theta band. Visual processing skills were crucial in allowing the infant to efficiently orient to the reward, and possibly predict its appearance. In order to successfully predict the reward appearance at the correct location, for each trial, participants would have to distinguish between the two flickering object’s frequencies, identify the higher vs. lower frequency and, over the course of the task, learn the association between the (higher vs. lower) frequency and reward appearance. Faster visual sampling and, therefore, higher visual temporal processing accuracy, would facilitate the distinction between the two object frequencies and the identification of the predictive frequency over the course of the task, translating into shorter saccadic latencies.

The three conditions (i.e., alpha, theta, mixed) can be viewed as different levels of difficulty. The mixed trials were the easiest, as they featured the highest difference in the two presentation frequencies (i.e., 2 Hz) among the three conditions. In alpha and theta conditions, while the absolute differences in the speed of the two presentation frequencies were approximately equal (~ 1 Hz), discerning between two alpha-band frequencies was more challenging than between two theta-band frequencies, as, according to Weber’s law, the discriminability between two magnitudes depends on the ratio, not the absolute difference, between their values^30^. Saccadic latencies to reach the reward were measured across trials and taken as a measure of visual temporal processing efficiency, with shorter saccadic latencies indicating more effective visual temporal processing.

Here, we tested the relation between PAF extracted from the EEG signal measured during resting state, and saccadic latencies to reach the reward across the three different within-participant conditions. Our hypothesis was that higher PAF would indicate faster visual sampling, shorter Temporal integration Windows and improved visual temporal processing. This would in turn result in better discerning of the two flickering frequencies, and therefore a quicker identification of the predictive frequency and faster attainment of the reward. Given the differing levels of difficulty among the three experimental conditions, we also tested potential variations in the relation between PAF and saccadic latencies across these conditions.

## Materials and methods

The study procedures and analyses reported here are a part of a larger project pre-registered on the platform AsPredicted [https://aspredicted.org/VM1_WMW] in line with open science practices.

### Participants

The participants were full-term, typically developing 6-month-olds (mean age = 199 days, standard deviation = 9 days). One hundred forty (*n* = 140) infants recruited from the database of Vienna Children’s Studies at the University of Vienna participated in the study, sixty-four of whom were male and seventy-six were female. The sample size was calculated based on estimated effect sizes for other effects investigated in this project (the discussion of which goes beyond the scope of the present manuscript). The study received approval of the Ethics Committee of the University of Vienna (ref. 00737). Before participation, informed written consent was obtained from the primary caregiver. The study was conducted in line with the principles of the Declaration of Helsinki.

Due to fussiness (*n* = 5), lack of enough data of sufficient quality (*n* = 26; see Sect. 2.2.5.2. for more details), or technical/experimenter errors (*n* = 11), EEG data was available for *n* = 98 participants. Looking behaviour data was available for *n* = 109 participants (*n* = 7 missing due to fussiness, *n* = 24 missing due to technical/experimenter errors). For *n* = 62 participants with looking behaviour data we were able to identify Peak Alpha Frequencies.

### Stimuli and procedure

#### Resting state

The participants viewed a non-social video^31^ for up to 2 min and 14 s. The video consisted of animated clips showing events such as leaves falling, a train moving, jellyfish swimming, and others. The presentation of the video was accompanied by white noise.

#### Predictive cueing task

**Fig. 1:**
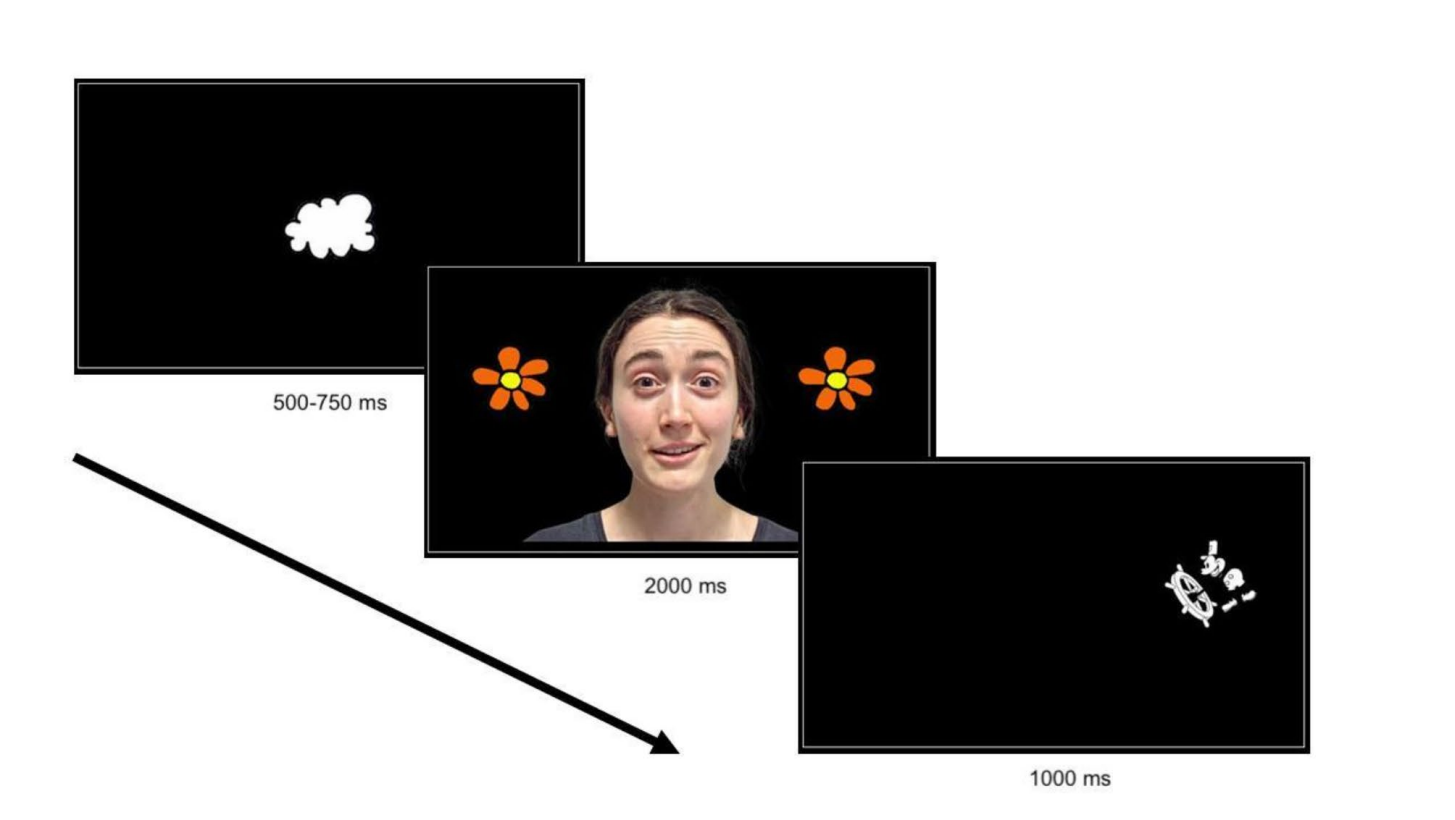
Predictive cueing task: trial structure.

In the task, infant attention shifts were cued bottom-up to either the right or the left side of the screen through the presentation of an animated character accompanied by a sound. At the beginning of the task trial, participants were presented with a fixation cue (cartoon cloud) for a varied randomised duration between 500 and 750 ms. Afterwards, a centrally presented face with direct gaze and two identical objects on the left and right side appeared on the screen for 2000 ms. This was accompanied by vocalisations such as “Wow, super!” or “Hello, baby!” (in German), to increase infant attention to the screen. The two identical objects flickered at two different frequencies: either both within the infant theta frequency band: 3.43 and 4.5 Hz (“theta” trials), both within the infant alpha frequency band: 5.54 and 6.55 Hz (“alpha” trials), or one within each frequency band: 4.5 And 6.55 Hz (“mixed” trials); the left-right side of the frequencies was counterbalanced within participants. After the 2000 ms, an animated character accompanied by a sound appeared for 1000 ms either on the right or the left side of the screen: for half of the participants always corresponding to the side of the lower, and for the other half – the higher frequency. Therefore, in each trial, to succeed in predicting the reward, one group of infants had to identify the lower flickering frequency as the predictive one, while the other group had to identify the higher flickering frequency. The task was designed to elicit steady-state visually evoked responses^32^ to covertly attended flickering stimuli (the analysis of which goes beyond the scope of the current manuscript). A schematic depiction of the trial structure of the task is shown in Fig. 1.

There were 16 trials per condition (48 trials in total), presented in a randomised order, with the only constraint being that no more than three trials of the same type could be presented in a row.

#### Procedure

The data collection took place at a designated Vienna Children’s Studies lab room. After explaining the study procedure and collecting informed consent from the caregiver, an EEG cap was placed on the infant’s head. The infant was seated on the caregiver’s lap, approximately 60 cm from the screen. After the recording of resting-state EEG, the participants were presented with two tasks: a Gaze-cue task not relevant to the present investigation (where infant attention was cued by gaze direction, counterbalanced across the side of the screen and the stimulus frequency within participants), and the Predictive cueing task, in a fixed order. It is important to note that in the Gaze-cue task, each frequency was paired with the gaze cue with equal probability within participants (counterbalanced across the trials), thereby preventing infants from learning any systematic gaze cue – frequency associations, and assuring no interference with the predictive cueing task. The tasks were presented on a Iiyama G-Master GB2560HSU screen with a 144 Hz refresh rate, and the accompanying audio was presented using Logitech Multimedia Z200 speakers. If the infant became fussy, the presentation of the tasks was paused and only resumed when the experimenters thought the infant was ready to continue. The entire procedure took approximately 10 min without breaks.

#### Looking behaviour data

Infant looking behaviour during Resting state and the Predictive cueing task was recorded using an Axis P1365 Mk II camera, at 25 frames per second. Offline coding of infant looking behaviour was later performed with the use of the Interact software (Mangold International GmbH, 2020). In the Resting state, infant gaze was coded as either directed towards the screen, or away from the screen. In the Predictive cueing task, infant gaze was coded as either directed towards the centre of the screen, towards the left side of the screen, towards the right side of the screen, or away from the screen. Out of the total of 140 videos, 25 videos were coded by two independent raters, frame by frame, yielding excellent inter-rater reliability (κ = 0.92).

##### Calculation of saccadic latencies

After the coding, data files containing looking behaviour codes were exported as .csv files and orientation latencies were then calculated using a custom script (written in Python, version 3.8.10), which first selects all trials during which the infant looks towards the centre of the screen during the reward onset, and then calculates the difference between the onset of the reward stimulus and the time of gaze orientation towards it. For the latency between stimulus appearance and gaze orientation to be included in our analyses, there were two conditions. Firstly, the orientation towards the location of the reward must have occurred before the onset of the fixation cue marking the end of the trial. Secondly, trials were only considered valid if no other looking behaviour was displayed between the central gaze and the orientation towards the location of the reward. For instance, if the stimulus appears laterally on the left-hand side, but the infant first looks right and then orients towards the stimulus, then the trial was considered invalid. No other exclusion criteria regarding the length of the saccadic latencies were applied. Since we also wanted to include anticipatory looks in our analyses, we included saccadic latencies < 100 ms - which is the threshold that in the literature is typically used to identify anticipatory saccadic movements^33^. On average, infants contributed 20.12 trials across conditions (range 1–41).

#### EEG

##### EEG data collection

The ActiCap EEG system (EasyCap GmbH) with 32 active Ag/AgCl electrodes was used in data collection. Conductive gel was injected into each electrode to ensure low impedances, which we aimed to keep below 10kΩ whenever possible. The online reference electrode was positioned behind the left ear (TP9), and the ground electrode was one of the frontal channels (Fp1). Otherwise, the layout followed the standard 10–20 system. One electrode was placed underneath the infant’s right eye with the purpose of capturing electrooculography (EOG) signal for tracking eye movements; this was often not well-tolerated by the infants, resulting in either missing or very noisy EOG data, which is why we decided not to include it in our analyses.

BrainProducts BrainAmp DC amplifier was used for EEG signal recording, with a sampling frequency of 500 Hz. The amplifier was connected to a BrainProducts TriggerBox device, which synchronised triggers sent from the presentation computer and the EEG/video recording computer. BrainVision Recorder (version 1.24.001, Brain Products GmbH, 2021) software was used for EEG signal recording.

##### EEG data preprocessing

The EEG signal was pre-processed with the use of the standardised HAPPILEE pipeline for lower density recordings^34^, in combination with custom MATLAB^35^ scripts, using the EEGLAB toolbox^36^. Firstly, line noise processing was conducted with a multi-taper regression approach to eliminate the 50 Hz electrical noise artefact. Next, a bandpass zero-phase Hamming-windowed sinc FIR filter with cut-off frequencies 1 and 100 Hz was applied to the continuous data. Next, artefact correction was performed with the use of wavelet thresholding. For the purpose of data rejection based on looking behaviour and signal quality, the signal was initially segmented into 1-second epochs. Channels deemed artefact-contaminated according to FASTER criteria (variance, median gradient, amplitude range, and deviation from mean amplitude^37^) were interpolated with spherical splines within epochs (up to 3 channels, otherwise the epoch was rejected). Finally, individual epochs were rejected based on a combination of amplitude-based (signal below/above +/- 150 µV) and joint-probability (considering how likely an epoch’s activity was given the activity of other epochs for that same channel, as well as other channels’ activity for the same segment) criteria. Indices of quality assessment of the pipeline performance were inspected, and participants for whom the cross-correlation values across all frequencies before and after wavelet thresholding were below 0.1 (indicating dramatic signal change during waveleting) were excluded from further analyses^38^. Data was then re-referenced to average reference. Finally, epochs where infants’ gaze was coded as directed away from the screen were rejected. Infants contributed an average of 64 clean segments (range: 15–91).

##### Spectral decomposition

Spectra analysis was performed on concatenated EEG segments from occipital (O1, Oz, O2) channels^25^. For each electrode, Power Spectral Density (PSD) was computed with the Welch method using the *pwelch* function in MATLAB (4 s Hanning window with 50% of overlap). Estimates of PSD for the three electrodes were averaged to obtain occipital spectral power.

##### Identification of PAF

**Fig. 2:**
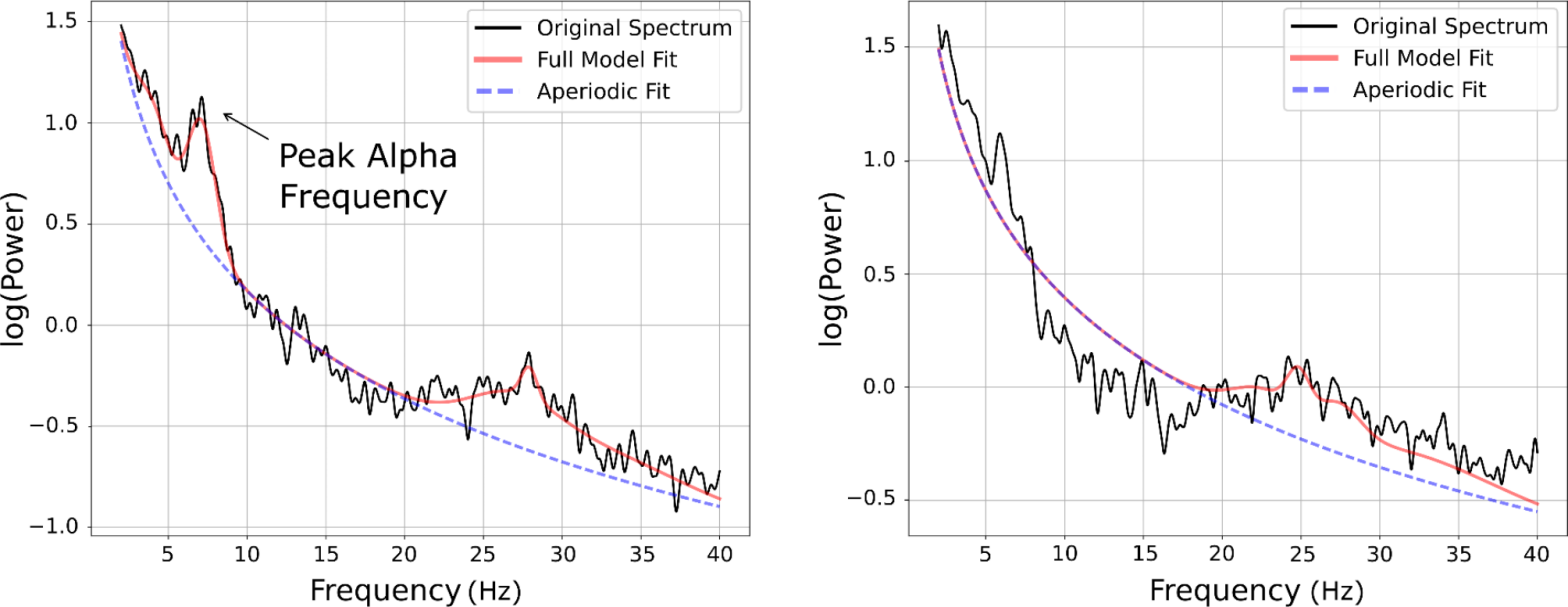
Illustration of the PAF extraction through the FOOOF algorithm. On the left a clear PAF identification is shown, while on the right a participant is shown for which FOOOF failed to identify a clear PAF.

To parameterize neural power spectra and extract PAFs, we used the fitting oscillations and one over f (FOOOF) algorithm^39^. The FOOOF algorithm allows a reliable estimation of peak frequencies above the aperiodic component (see Fig. 2). Input parameters for the algorithm to identify the peaks were: peak width limits: 1.0–10.0; max number of peaks: 5; minimum; peak height: 0.2; peak threshold: 1.85; and aperiodic mode: fixed. In selecting these parameters, we initially relied on the work of Leno et al.,^25^. However, since our sample differed from theirs in terms of age, and Leno et al.^25^’s parameters resulted in underfitting our power spectral densities (PSDs), we implemented the adjustments recommended in the FOOOF tutorial^39^ and, through visual inspection of model fits, we identified parameters that provided the closest match to our raw PSDs (refer to Fig. 2 for examples of FOOOF outputs with and without a distinct alpha peak). Power spectra were parameterized across the frequency range 2–40 Hz. Only participants for whom FOOOF identified peaks in the 5–12 Hz range were included in the analyses. We selected the 5–12 Hz range based on previous works suggesting that this frequency range is appropriate for extraction of PAF at this age^2^. According to the FOOOF algorithm, 18 participants showed two peaks in the 5–12 Hz range. Following Leno et al.,^25^, for these participants the higher value was considered as their PAF. Out of 98, for 64 participants (65.3%) it was possible to identify PAF through FOOOF. The success rate is relatively lower than the 80% obtained in the study by Leno et al. (2021), but expected given the younger age of our participants, and the developmental trends in the visibility of peak frequencies^40^. The mean of the goodness of FOOOF fits was *R*^*2*^ = 0.97, with a mean error of 0.06.

## Results

Statistical analyses were conducted in the R environment (R Core Team, 2021), with linear mixed-effects models performed with the lme4^41^ and lmerTest^42^ packages. Descriptive statistics of our main variables of interest are shown in Table 1.

**Table 1:** Saccadic latencies (ms) and number of valid trials across condition and in the three different conditions of the predictive cueing task.

	*n*	Mean	SD	Minimum	Maximum
Average latency across conditions [ms}	109	401	120	120	668
Average latency in the theta condition [ms]	108	391	130	121	706
Average latency in the mixed condition [ms]	106	404	132	87	757
Average latency in the alpha condition [ms]	106	406	126	144	663
Peak Alpha Frequency [Hz]	64	6.93	1.39	5.09	11.60
Average n. of valid trials across conditions		19.77	10.58	1	41
Average n. of valid trials in the theta condition		6.65	3.67	1	16
Average n. of valid trials in the mixed condition		6.79	3.38	1	14
Average n. of valid trials in the alpha condition		6.65	4	1	16

**Fig. 3:**
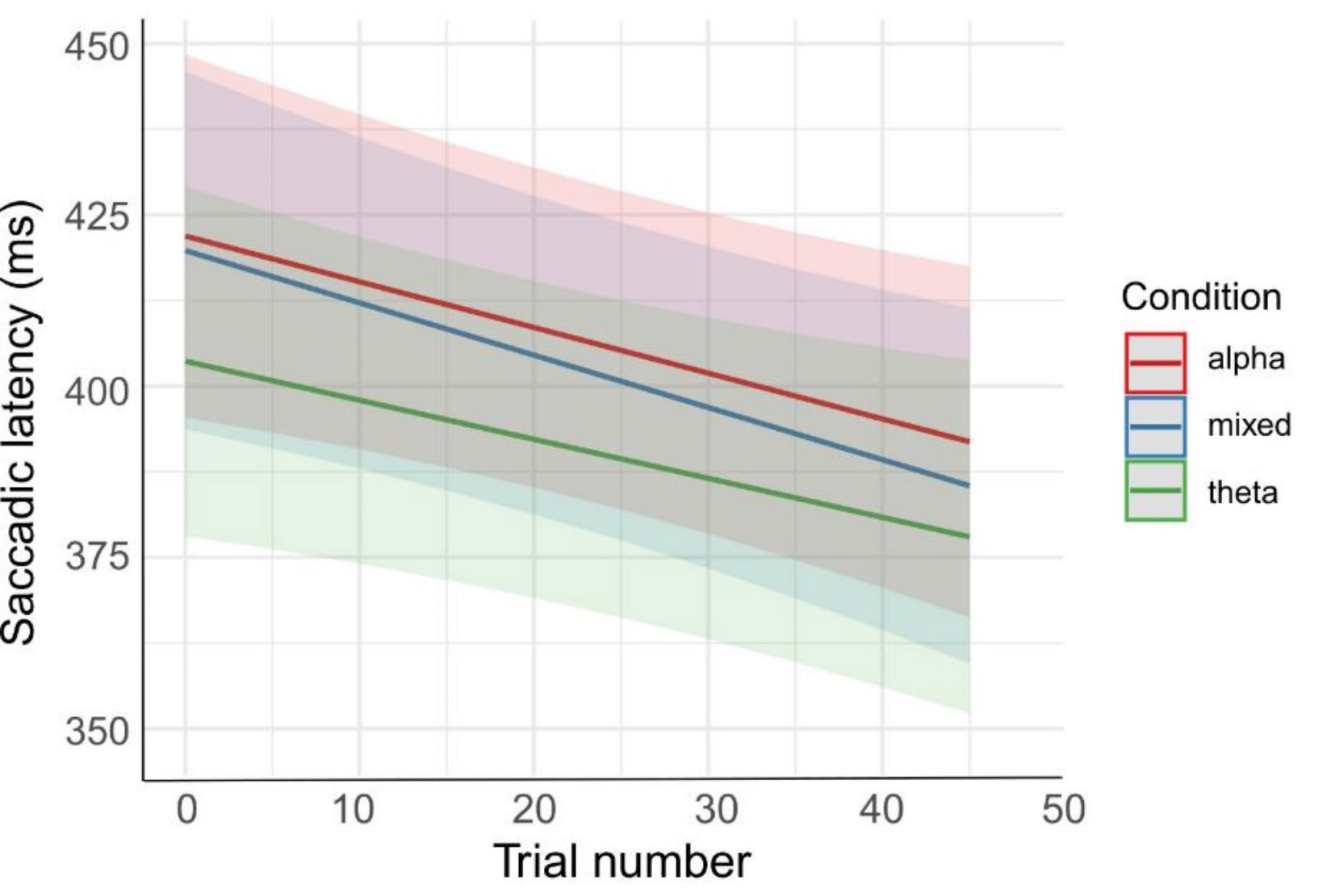
Infants’ saccadic latencies in the three conditions over the course of the trials. The three coloured lines represent the saccadic latency’s trends across trials in the three different conditions. The coloured areas represent the confidence intervals (0.95).

To test whether infants learned the association between the predictive frequency and the reward appearance, we ran a linear mixed model with log-transformed Saccadic latency as dependent variable, Trial as fixed predictor and random intercept for participant. Model results (*marginal R*^*2*^ = 0.003; *conditional R*^*2*^ = 0.58) showed that saccadic latencies decreased over the course of the trials *F*(1, 2111.6) = 15.09, *p* < .001 (Fig. 3).

To analyse the relation between PAF and saccadic latencies across the three conditions, log-transformed saccadic latencies^43^ to reach the reward were entered in three linear mixed models: one (model1) including PAF, Condition (alpha, theta, mixed) and their interaction as fixed predictors; the second (model2) including PAF, Condition and Frequency (3.43, 4.5, 5.54, 6.55 Hz) and their interactions as fixed predictors; and the third (model3) with PAF, Condition, Group (High Predictive Frequency vs. Low predictive frequency) and their interactions as fixed predictors. All the models also included by-subject random intercept. Models 2 and 3 were run to explore possible effects of the specific predictive frequency. Model comparison was performed by calculating the Akaike information criterion (AIC^44^) of the three models. AIC provides an estimate of model quality, with lower values indicating better models. A ΔAIC = 2 typically suggests evidence in favour of the model with the lower AIC^45^. According to AIC, model1 resulted to be the best model (AICmodel1: 925.54; AICmodel2: 960.91; AICmodel3: 957.73), thus suggesting that the specific predictive frequency or the group to which infants were assigned were not influential factors in this analysis.

**Fig. 4:**
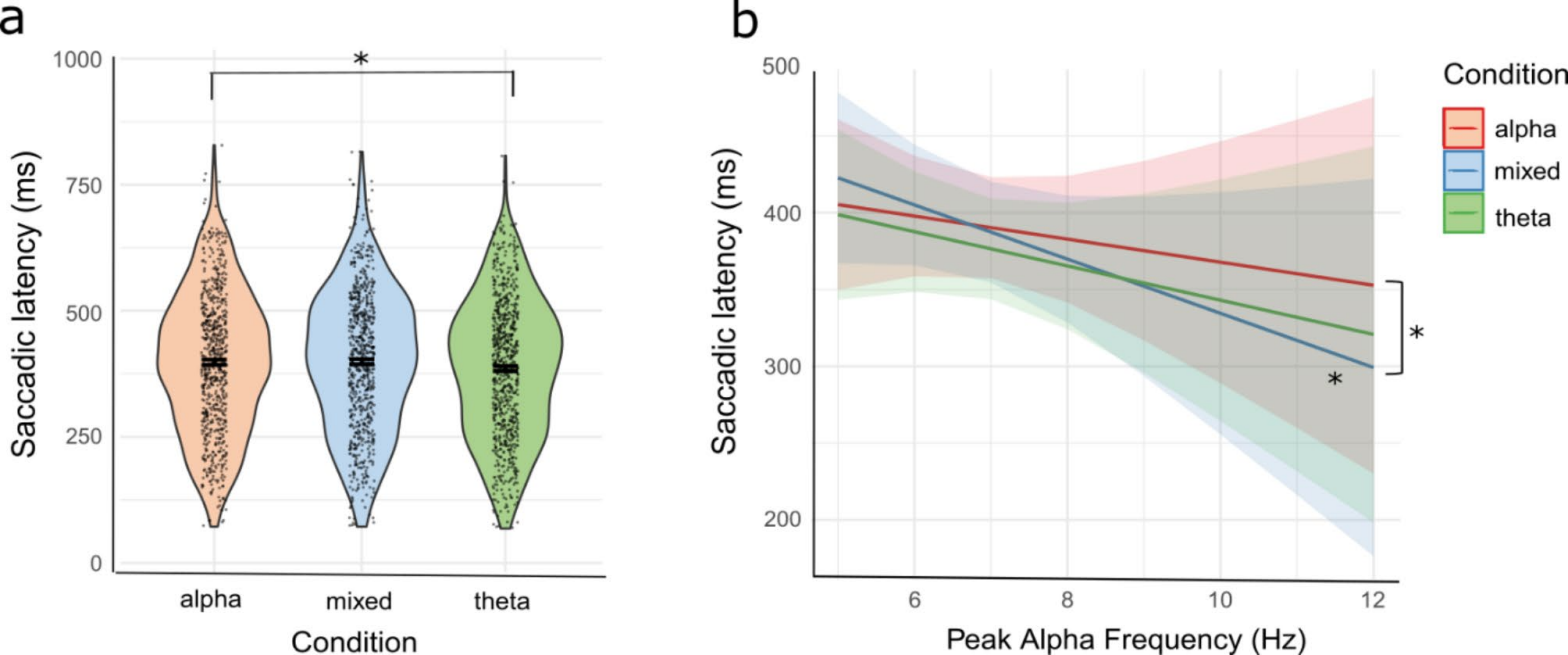
Panel (**a**) displays the saccadic latencies across alpha, theta and mixed conditions. Dots represent individual trial saccadic latencies, while the segments within each violin plot show the mean of the saccadic latencies in each condition. Panel (**b**) shows saccadic latency trends in the three different conditions depending on participants’ PAF. Coloured areas represent confidence intervals for each condition. * *p* ≤ .05.

Model results (*marginal R*^*2*^ = 0.02; *conditional R*^*2*^ = 0.6) revealed a main effect of Condition, *F*(2, 1251.6) = 3.81, *p* = .022, with Bonferroni-corrected post-hoc comparisons showing that infants display shorter saccadic latencies in theta trials (*M* = 377 ms, *SE* = 16.7) compared to alpha trials (*M* = 390 ms, *SE* = 16.7), *t*(1252) = 2.63, *p* = .026 (Fig. 4, panel a). Additionally, we found a significant interaction between PAF and Condition, *F*(2, 1251.31) = 3.95, *p* = .019 showing that higher PAF is associated with shorter saccadic latencies in mixed trials, t(66.7) = 1.94, *p* = .05, and that the linear trend observed for the mixed trials (*PAF trend* = − 0.07, *SE* = 0.04) significantly differed from the one observed in the alpha trials (*PAF trend* = − 0.03, *SE* = 0.04) (Fig. 4, panel b). No other significant differences were found (all *ps* > 0.23). Additional analyses were conducted separately for saccadic latencies below and above 100 ms latencies, revealing that excluding the latencies below 100 ms does not affect the results. Moreover, we also tested whether there were differences in behavioural performance or demographics between infants having vs. not having a clear PAF as estimated by FOOOF; results revealed no significant effects (see Supplementary Materials for detailed analyses).

Lastly, to explore whether the shortening of saccadic latencies with the increase of PAF in the mixed condition becomes more evident as the trials progress - in other words, to explore whether infants progressively learn the association between the higher vs. lower frequency and the reward appearance over the course of the task in the mixed condition and if the speed of learning varied as a function of PAF - we run an additional analysis, entering log-transformed saccadic latencies in a linear mixed model with PAF, Condition and Trial number, and their interactions as fixed predictors and a random intercept for participant. Results from this model (*marginal R*^*2*^ = 0.03; *conditional R*^*2*^ = 0.6) revealed the same effects found in the previous analysis: a main effect of Condition, *F*(2, 1247.47) = 3.06, *p* = .047, and PAF x Condition interaction, *F*(1, 1252.06) = 0.45, *p* = .026. The three-way interaction was not significant *F*(2,1246.56) = 2.31, *p* = .099 and no other main effects or interactions were found (all *ps* > 0.12).

## Discussion

A growing body of studies has provided evidence that the temporal resolution of adults’ visual attention is closely related to the frequency of alpha-band oscillations, with higher Peak Alpha Frequency being associated with better visual temporal processing skills. However, no studies to date have tested whether the same relationship occurs in infancy. Our study investigated - for the first time - the role of Peak Alpha Frequency in the development of visual temporal processing in early infancy. To this end, we tested the relationship between PAF extracted during a resting state EEG recording and saccadic latencies in a predictive cueing task in 6-month-olds. As the performance in the predictive cueing task relied on visual temporal skills (better discrimination of the two frequencies and identification of the predictive frequency), we hypothesised that higher PAF would predict shorter saccadic latencies, with possible differences across the three conditions.

Our results showed that infants with higher PAF displayed shorter saccadic latencies in the mixed condition, and that this trend differed from the one found in alpha trials, where this relation did not occur. Thus, having higher PAF did not translate into shorter saccadic latencies overall, but the effect was specific to one condition. The fact that the relation between PAF and saccadic latency occurs only in the mixed condition could be explained by the fact that, in terms of frequency discrimination, the mixed trials are easier compared to the alpha and theta ones, as the difference between the two object frequencies is higher in the former condition (2 Hz) compared to the other two (1 Hz). This might have facilitated the distinction between the two flickering frequencies, and the identification of the predictive frequency of the reward for each trial. Therefore, at this age, it is possible that the facilitating effects of having a higher PAF might emerge only in conditions where visual temporal processing demands are not too high.

Our results align with the idea that the frequency of alpha-band oscillations is implicated in establishing the temporal resolution of visual attention^15^, determining its sampling rate, and demonstrate that this happens already at early stages of life. Indeed, we showed that, already at 6 months, the interindividual variability in PAF is linked to infants’ ability to reach the reward in a predictive cueing task where visual temporal skills were crucial, suggesting that the pace of alpha-band oscillations influences the temporal resolution and accuracy of visual experience already in early infancy. In other words, higher PAF would allow our brain to sample the environment more frequently, resulting in a more faithful representation of the external environment and, in our task, higher discriminative capacity between the two predictive flickers.This, in turn, would facilitate the learning of the association between the higher vs. lower frequency and the reward appearance. Although we did not observe evidence of a steeper learning in the mixed condition as a function of PAF – possibly due to the low number of trials per condition (refer to Table 1 for details) - it is worth noting that overall saccadic latencies decrease over the course of the task. This finding suggests that infants progressively learn the association between the predictive frequency and the reward appearance, leading to a linear decrement of the saccadic latencies to reach the reward.

Our results also fit with the observation that the developmental trajectory of PAF mirrors the one of visual temporal processing in the first years of life^3^, corroborating the conceptualization of PAF as a driver of visual temporal processing development. The shortening of visual temporal integration windows with development might also reflect a broader, domain-general process of narrowing of temporal precision in environment sampling^46^. Thereupon, future research should examine the role of PAF in multisensory (e.g., visual-auditory) integration, and perceptual narrowing effects.

A methodological strength of this study is that our estimation of PAF was implemented with the use of a method which takes into consideration the aperiodic component (1/f-like) of the neural power spectra. Electrophysiological neural activity is usually analysed without considering this component and this might lead to conflating periodic parameters (center frequency, power, bandwidth) with aperiodic ones (offset, exponent), then compromising physiological interpretations^39^. By parameterizing both periodic (i.e., oscillatory) and aperiodic (i.e., arrhythmic, 1/f-like) components, this approach increases analytical power and allows a reliable estimation of periodic parameters. Using this method, analyses revealed that in the current sample of 6-month-old infants, the average PAF was 6.98 Hz, in line with previous works investigating alpha-band oscillations in infancy^2,40^.

Another interesting result of our study was that infants’ overall saccadic reaction times were shorter in the theta condition compared to the alpha condition. This finding is consistent the idea that alpha and theta brain rhythms differently contribute to attentional processes^47,48^ while alpha-band activity is linked to visual attention sampling processes^24^, theta band-oscillations are involved in spatial attention and exploration processes^49–52^. Indeed, it has been shown that in tasks where exploration of the visual space is required (e.g., visual search tasks, attentional reorienting tasks), attention reorients periodically at ∼4 Hz (theta) between the two stimulus locations but samples each location periodically at ∼11 Hz (alpha)^48^. In line with this perspective, a number of studies have provided evidence of the involvement of the theta brain rhythm in spatial attention mechanisms during early development. For instance, Bache et al.,^53^ showed that 10-month-old infants’ theta brain activity was particularly pronounced during visual tracking of movements. Similarly, theta brain oscillations increase during focused exploration of novel objects in 11-month-olds^54^. As evidence exists that already in infancy periodic presentation of visual stimuli may entrain endogenous rhythms at corresponding frequencies^32^, it is possible that in our study, entrainment of infants’ brain activity to the theta rhythm promoted the attentional shifts and made infants faster in reaching the reward. Although further investigation is needed to empirically test this hypothesis, this result further supports the proposed role of the theta rhythm in visuospatial attention in early infancy.

Hence, it is possible that in our predictive cueing task, both bottom-up and top-down processes contributed to reducing saccadic latency to the reward. On the one hand, in the theta trials, entrainment to theta rhythm might have facilitated attentional shifts. On the other hand, in the mixed trials, PAF promoted better temporal resolution of visual attention, leading to higher discriminative capacities and the learning of the reward’s location. While our task was designed to capture several cognitive processes, we argue that PAF should also predict infant performance in simpler tasks relying on visual temporal attention. Future research should examine the relation between PAF and infant performance in other attentional tasks, to further probe the role of PAF in visual temporal sampling of the environment.

Our findings have important implications from a clinical and therapeutic point of view. The adverse impacts of disruptions in visual temporal processing are now widely recognized. Dysregulation at an early stage of the perceptual hierarchy can lead to a cascade effect in later sensory and cognitive processes, as higher nodes in the perceptual chain rely on a compromised signal for their computations. Evidence in this direction comes from studies on neurodevelopmental conditions such as autism, where high PAF and the attunement to fast-paced visual events may contribute to the observed predominance toward bottom–up versus top–down processing observed in autistic individuals^29,55,56^. Another example of disruptive visual temporal processing is dyslexia, often associated with difficulties in segmenting the visual environment, thereby compromising reading skills^9^. These considerations underscore the importance of identifying early intervention strategies aimed at boosting infants’ temporal accuracy of visual attention. Recent research has already proved the efficacy of neurostimulation interventions^57,58^ as well as sensory entrainment^19,59–61^ in successfully shaping adults’ alpha frequency. Implementing interventions that target visual temporal processing by leveraging Peak Alpha Frequency in the early stages of development could trigger a domino effect on various socio-cognitive abilities, laying a foundation to future clinical applications and applied neuroscience approaches.

To sum up, we have provided evidence supporting the idea that starting from very early stages of life, the frequency of alpha-band oscillations is involved in the temporal organisation of visual attention. Specifically, 6-month-old infants with higher PAF were quicker to orient to the reward appearance, likely due to their advantage in distinguishing and identifying higher vs. lower visual predictive flicker. Our findings provide novel insights into the neural correlates and early mechanisms of infants’ visual temporal and spatial attention, which will hopefully inform future research on supporting infants in accurate perception of their visual environment.

## Electronic supplementary material

Below is the link to the electronic supplementary material.

Supplementary Material 1

## Data Availability

The data and analysis scripts that support the findings of this study and supplementary materials are openly available in OSF at https://osf.io/cx7hb/.
