# Supplementary material for: Peak alpha frequency is linked to visual temporal attention in 6-month-olds
Source: Sci Rep. 2024 Nov 15;14:28173. doi: 10.1038/s41598-024-79129-0 (PMC11568323; doi:10.1038/s41598-024-79129-0)
Supplement: Supplementary file 1 — Supplementary Material 1 [file 41598_2024_79129_MOESM1_ESM.docx]

**Supplementary Materials**

**1.** **Analysis on saccadic latencies > 100 ms (N=110; obs = 2150)**

Saccadic latencies higher than 100 ms were log-transformed ^40^ and entered in a linear mixed model including PAF, Condition (alpha, theta, mixed) and their interaction as fixed predictors and by-subject random intercept. Model (*marginal R^2^* = .02; *conditional R^2^* = .61) revealed the same pattern of results of the model that also included the saccadic latencies < 100 ms: a main effect of Condition, *F*(2, 1225.26) = 3.78, *p* =.023, and a significant interaction between PAF and Condition, *F*(2, 1224.99) = 3.86, *p* = .021.

**2.** **Analysis on saccadic latencies < 100 ms (N=21; obs = 45)**

Saccadic latencies lower than 100 ms were log-transformed ^40^ and entered in a linear mixed model including PAF, Condition (alpha, theta, mixed) and their interaction as fixed predictors and by-subject random intercept. Model (*marginal R^2^* = .055; *conditional R^2^* = .055) revealed no significant effects (all *ps* > .83). It is worth considering that the small number of observations may not provide sufficient power to detect meaningful effects. Further investigation with a larger sample size might be needed to draw more definitive conclusions.

**3. Analysis including only IDs with number of EEG segments > 1**

Saccadic latencies for all the IDs contributing with more than 1 trial were included in this analysis. Log-transformed ^40^ saccadic latencies were entered in a linear mixed model including PAF, Condition (alpha, theta, mixed) and their interaction as fixed predictors and by-subject random intercept. Model (*marginal R^2^* = .025; *conditional R^2^* = .60) revealed the same pattern of results of the model that also included the saccadic latencies < 100 ms: a main effect of Condition, *F*(2, 1251.60) = 3.81, *p* =.022, and a significant interaction between PAF and Condition, *F*(2, 1251.31) = 3.95, *p* = .019.

**4. Comparison of behavioural performance between infants for whom FOOOF identified vs. did not identify a clear PAF**

To test whether there were any behavioural performance differences between infants for which FOOOF identified vs did not identify a clear PAF (64 vs.47 IDs, respectively), we run two linear mixed models: model1 had saccadic latency as DV, PAF Group (yes, no), Condition and their interaction as fixed predictors and random intercept for participants; model2 had saccadic latency as DV, PAF Group (yes, no), trial number and their interaction as fixed predictors and random intercept for participants. Results showed that having vs. not having a clear PAF in the PDSs did not affect saccadic latencies across conditions (model1) and trials (model2) (all *ps* > .14) (Figure S1).


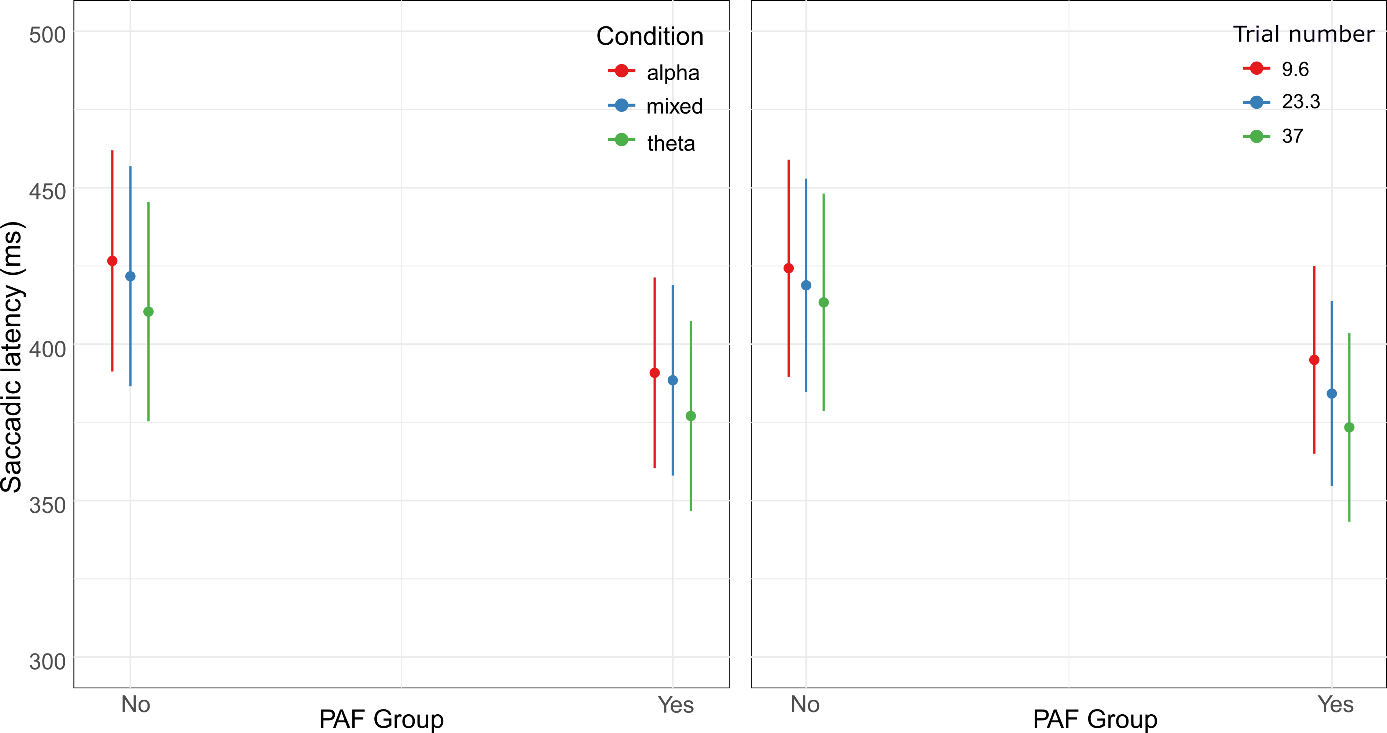


**Figure S1**. Infants’ saccadic latencies across trials and conditions in the two groups of infants for whom FOOOF identified vs. did not identify a clear PAF.

**5. Comparison of demographic variables between infants for whom FOOOF identified vs. did not identify a clear PAF**

To test whether there were any demographic differences between infants for which FOOOF identified vs did not identify a clear PAF (64 vs.47 IDs, respectively), we run a series of chi-squared tests between PAF Group (yes, no) and gender (female, male), education level of the primary and secondary caregivers (university, higher vocational school, academic secondary school, apprenticeship), household income, cultural affiliation of primary and secondary caregivers, birth country of primary and secondary caregivers, number of people living in the same house of the baby. No significant effects were found, (all *ps* > .18). Moreover, we also run four separate logistic regressions with PAF group (yes, no) as dependent variable and age of primary and secondary caregivers, months of breastfeeding and months of nursery attending as predictors. No significant effects were found, (all *ps* > .28).
